# Speicheldrüsenultraschall oder Biopsie? Methoden im Vergleich anhand von Fallbeispielen

**DOI:** 10.1007/s00393-023-01416-4

**Published:** 2023-10-02

**Authors:** J. Peters, S. Timme-Bronsert, R. E. Voll, S. Finzel

**Affiliations:** 1 Institut für Klinische Pathologie, Universitätsklinikum Freiburg und Medizinische Fakultät, Universität Freiburg, Hugstetter Str. 55, 79106 Freiburg, Deutschland; 2 Klinik für Rheumatologie und Klinische Immunologie, Universitätsklinikum Freiburg und Medizinische Fakultät, Freiburg, Deutschland; 3 Klinik für Rheumatologie und klinische Immunologie, Universitätsklinikum Freiburg, Hugstetter Str. 55, 79106 Freiburg, Deutschland

**Keywords:** Sjögren-Syndrom, Immunglobulin G4, Sarkoidose, Histologie, Sicca-Syndrom, Sjögren‘s syndrome, Immunoglobulin G4, Sarcoidosis, Histology, Sicca syndrome

## Abstract

**Hintergrund:**

Der Ultraschall der Speicheldrüsen (SD) ist eine schnell durchführbare und nichtinvasive Methode, Sjögren-Syndrom(SS)-typische Veränderungen der großen SD zu detektieren und semiquantitativ einzuschätzen. Die Differenzialdiagnose des SS ist komplex, da zahlreiche Krankheiten und Therapienebenwirkungen ein dem SS ähnliches klinisches Bild mit Sicca-Syndrom und z. T. Speicheldrüsenveränderungen verursachen können („Nachahmer-Erkrankungen“). Lange Zeit galt daher die SD-Biopsie, besonders bei SS-A-negativen Patienten, als Diagnostik der Wahl, während der Stellenwert der SD-Sonographie auch heute noch kontrovers diskutiert wird.

**Ziel der Arbeit:**

Es erfolgt eine Gegenüberstellung SS-typischer und -untypischer Veränderungen der Speicheldrüsen im Ultraschall und dazugehöriger histologischer Schnitte.

**Material und Methoden:**

Sechs Patientenfälle mit Antikörper-positivem bzw. -negativem SS mit und ohne SS-typischen Ultraschallbefund, SS-assoziiertem Lymphom, Sarkoidose sowie IgG4-assoziierter Erkrankung werden beschrieben. Die Befunde der sonographischen Untersuchung der Parotisdrüsen sowie die dazugehörige Histologie der SD werden erläutert und in Kontext gesetzt.

**Ergebnisse:**

SS-A-Antikörper-positive Patienten mit SS weisen v. a. bei länger bestehender Erkrankung ein typisches sonographisches Muster mit echoarmen Foci auf. Dieses Muster kann die Diagnose eines SS untermauern helfen. Die Ultraschallmuster der Nachahmer-Erkrankungen unterscheiden sich teils deutlich von primäres Sjögren-Syndrom(pSS)-typischen Mustern. Die histologische Untersuchung der SD hilft bei der Diagnosefindung, jedoch bedürfen gerade niedrige histologische Fokus-Scores einer kritischen Zusammenschau der klinischen, serologischen und bildgebenden Befunde.

**Diskussion:**

Sowohl der Speicheldrüsenultraschall als auch die histologische Aufarbeitung der Speicheldrüsenbiopsien haben eine Berechtigung in der SS-Diagnostik und Differenzialdiagnose des Sicca-Syndroms.

## Abstract (en)

**Background:**

Ultrasound examination of the salivary glands (SG) is a quick and noninvasive method to detect and semiquantitatively estimate typical changes in the large SG in Sjögrenʼs syndrome (SS). The differential diagnosis of SS is difficult because several diseases and adverse effects of treatment have a similar clinical picture as SS with sicca syndrome and can even induce alterations in the SG (mimic diseases). Hence, for a long time an SG biopsy was regarded as the diagnostic procedure of choice, especially in SS‑A negative patients, whereas the significance of SD sonography is still controversially discussed.

**Objective:**

Comparison of typical and atypical changes for SS in the salivary glands in ultrasound and associated histological sections.

**Material and methods:**

This article describes six patient cases with antibody positive or negative SS with and without typical SS ultrasound patterns, SS-associated lymphoma, sarcoidosis and IgG4-associated disease. The findings of the sonographic examination of the parotid glands and the associated histology of the SD are explained and put into context.

**Results:**

The SSA antibody positive patients with SS show a typical sonographic pattern with hypoechoic foci, especially if the disease has been present for a long time. This pattern can help support the diagnosis of SS. The ultrasound patterns of the mimic diseases sometimes differ significantly from the typical patterns of pSS. The histological examination of the SG helps to corroborate the diagnosis but low histological focus scores, in particular, require a critical synopsis of the clinical, serological and imaging findings.

**Conclusion:**

Both salivary gland ultrasound and the histological examination of SG biopsies are justified in the diagnostics and differential diagnosis of SS and sicca syndrome.

Der Ultraschall der Speicheldrüsen (SD) ist eine schnell durchführbare und nichtinvasive Methode, Sjögren-Syndrom(SS)-typische Veränderungen der großen SD zu detektieren und zu quantifizieren. Die Differenzialdiagnose des SS wird durch zahlreiche „Nachahmer“-Erkrankungen erschwert, die ein ähnliches klinisches Bild einschließlich sonographisch fassbarerer SD-Veränderungen induzieren können. In unklaren Fällen wurde daher die Speicheldrüsenbiopsie als das diagnostische Verfahren der Wahl angesehen, während die Bedeutung des sonographischen SD-Diagnostik z. T. noch kontrovers diskutiert wird.

Das Sjögren-Syndrom (SS) gehört zu den Kollagenosen und ist eine chronisch entzündliche Autoimmunerkrankung. Die genaue Genese des SS ist bis dato noch nicht vollständig geklärt. Es wird jedoch vermutet, dass autoreaktive T‑ und B‑Zellen sowie nukleäre Antikörper, wie beispielsweise Anti-SS-A-Antikörper, gerichtet gegen das Endothel der Ausführungsgänge exokriner Drüsen (wie Parotis und Submandibularisdrüsen), zu einer dauerhaften Entzündung und hierüber allmählich zu einer Fibrosierung und schließlich einem Funktionsverlust der betroffenen Organe führen [13]. Diese Umbauvorgänge führen zu strukturellen Veränderungen in den Speicheldrüsen, die sich mittels bildgebender Verfahren, die auch den Speicheldrüsenultraschall (SDUS) umfassen, schnell und nichtinvasiv darstellen lassen [1].

In den letzten Jahren hat die Ultraschall Subtaskforce der Outcome Measures in Rheumatology Clinical Trials (OMERACT) zunächst eine gemeinsame Definition für SDUS im B‑Bild entwickelt, um SS-typische Schädigungsmuster quantifizieren zu können [8], und danach selbiges auch für den Color-Doppler, um Hypervaskularisierung zu quantifizieren, die als Zeichen der Krankheitsaktivität gilt [10]. Anschließend wurden die Definitionen und dazugehörigen Ultraschallscores an Patientenkohorten in „reliability exercises“ validiert [8, 10].

Hypervaskularisierung gilt als Zeichen der Krankheitsaktivität

Typische SDUS-Veränderungen im B‑Bild erscheinen als unterschiedlich ausgeprägte Inhomogenitäten der Drüse, zusammen mit fokalen echoarmen oder echofreien Bereichen. Grad 0 bezeichnet sonographisch unauffällige Speicheldrüsen, Grad 1 zeichnet sich durch leichte Inhomogenität ohne echoarme oder echofreie Bereiche und echoreiche Bänder aus, Grad 2 durch mäßige Inhomogenität mit fokalen echoarmen oder echofreien Bereichen, Grad 3 durch typische, echoarme Foci, entsprechend einer schweren Inhomogenität mit diffusen echoarmen oder echofreien Bereichen, alternativ Vorliegen einer fibrös umgebauten Drüse mit hyperechogenen Bändern, die die gesamte Oberfläche der Drüse bedecken, was die Drüse dem angrenzenden Gewebe ähneln lässt [8].

In ähnlicher Weise graduiert der Color-Doppler-Ultraschall die Ausdehnung des Areals mit vermehrter Durchblutung, das als Hypervaskularisierung angezeigt wird. Niedrigere Scores entsprechen dabei keiner bis geringer Krankheitsaktivität und umgekehrt (Grad 0 = keine sichtbaren Gefäßsignale, Grad 1 = fokale, verstreute Gefäßsignale, Grad 2 = diffuse Gefäßsignale in < 50 % der Drüse, Grade 3 = diffuse Gefäßsignale in > 50 % der Drüse) [10].

In einer frühen Studie konnten Jousse-Jouline et al. zeigen, dass der SDUS sensitiv für Veränderungen unter Therapie ist [11]. Daher wird der SDUS zunehmend als Outcome-Tool in klinischen Studien eingesetzt [6] oder kann als hochsensitive Bildgebungsmethode dazu dienen, eine Verdachtsdiagnose zu untermauern [17]. Künftig könnte der SDUS sogar unterschiedliche SS-Phänotypen morphologisch helfen zu identifizieren [12]. Dennoch ist der SDUS nach wie vor noch nicht Bestandteil diagnostischer internationaler Leitlinien [4], gewinnt jedoch zunehmend an Akzeptanz [16, 19], auch, da sich die Veränderungen sehr sensitiv und spezifisch mittels des OMERACT-Scores quantifizieren lassen [7].

Die Speicheldrüsenbiopsie (SDB) hat nach wie vor einen hohen Stellenwert in der Diagnostik und Differenzialdiagnostik des SS, wird jedoch nicht selten von den zu Untersuchenden abgelehnt aus Angst vor Schmerzen oder Nervenverletzungen. Aus Erfahrung im eigenen Zentrum lässt sich berichten, dass die Akzeptanz der Biopsie der kleinen Speicheldrüsen dabei höher ist und mit weniger Bedenken belegt als die operative Biopsie der großen Speicheldrüsen. Die SDB ist als Bestandteil der EULAR(European League Against Rheumatism)-Klassifikationskriterien nach Shiboski et al. mit einem hohen Punktescore bewertet, gleichauf mit der Positivität für SS-A-Antikörper [14].

Im Jahr 1963 beschrieben Waterhouse et al. erstmals ein Fokussystem zur Festlegung des Schweregrades einer Speicheldrüsenadenitis. Das fünfstufige Fokussystem basierte auf dem Ausmaß der lympho- und histiozytären Infiltration in 4 mm^2^. Dabei entsprach ein Fokus einem Aggregat von 50 oder mehr Lympho- und Histiozyten [21]. Im Jahr 1968 etablierten Chisholm und Mason mit dem Fokus-Score ein fünfstufiges Gradingsystem, in dem sowohl das Ausmaß der lympho- und histiozytären Infiltration als auch das Vorliegen (mindestens) eines Fokus einbezogen wurden. Dabei entspricht Grad 0 der Abwesenheit eines Entzündungszellinfiltrates, Grad 1 einer geringen chronischen Entzündung, Grad 2 einer mäßigen chronischen Entzündung, die allerdings nicht die Kriterien eines Fokus erfüllt, Grad 3 dem Vorliegen von einem Fokus, Grad 4 dem Vorliegen mehrerer Foci [3]. Ergänzend wurde für Proben, in denen das Ausmaß der lympho- und histiozytären Infiltration so stark war, dass einzelne Foci nicht mehr voneinander abgrenzbar sind, ein Focus-Score 12 eingeführt. Trotz weiterer Versuche, durch ergänzende histologische Kriterien die Sensitivität und Spezifität der histologischen Sjögren-Diagnostik zu erhöhen [5, 9, 18], ist der Chisholm- und Mason-Fokus-Score weiterhin das in der Routinediagnostik am weitesten verbreitete Scoringsystem.

Dennoch ergibt die SDB bei Weitem nicht immer wegweisende Ergebnisse, und es bedarf zusätzlicher Färbungen [20] bzw. einer guten Präselektion der zu biopsierenden Patient*innen [2]. Mitunter führt jedoch erst die Zusammenschau aus Anamnese, Klinik, Serologie, Histologie und bildgebenden Verfahren schließlich zur korrekten Diagnose.

Der vorliegende Beitrag dient dazu, die Vor- und Nachteile beider Methoden anhand von Fallbeispielen weiter herauszuarbeiten und verständlich zu machen.

## Material und Methoden

### Beschreibung Patientin 1

Die ersten Beschwerden der 32-jährigen Patientin hatten bereits 15 Jahre vor Erstdiagnose mit okulärer und oraler Sicca-Symptomatik begonnen. Binnen 3 Jahren vor Diagnose waren Arthritis der kleinen proximalen Interphalangeal‑, Hand‑, Knie- und Sprunggelenke hinzugekommen mit Morgensteifigkeit bis zu 2 h. Zudem berichtete die Patientin über rezidivierende Schwellungen der großen SD, der zervikalen und nuchalen Lymphknoten und eine ausgeprägte Fatigue. Die genannten Befunde bestätigten sich bei Vorstellung in der Ambulanz, es bestand zudem eine limitierte, palpable Purpura an beiden Unterschenkeln.

### Beschreibung Patientin 2

Die 56-jährige Patientin stellte sich mit ausgeprägter okulärer und oraler Sicca-Symptomatik zur Zweitmeinung vor. Extern war ein seronegatives SS seit 2 Jahren vermutet worden, die Sicca-Symptomatik bestand seit 11 Jahren. Die Patientin berichtete über gelegentliche Arthralgien ohne Morgensteifigkeit oder Gelenkschwellungen sowie über intermittierende Lymphknoten- und SD-Schwellungen. Klinisch ließen sich die Parotisdrüsen (PD) mit 3 cm und die Submandibularisdrüsen (SMD) mit 2 cm leicht vergrößert palpieren, der Zahnstatus war reduziert. Zur weiteren Abklärung führten wir einen SDUS sowie eine Lippenspeicheldrüsenbiopsie durch.

### Beschreibung Patientin 3

Die 71-jährige Patientin wurde uns zur Mitbeurteilung vorgestellt, extern war der Verdacht auf ein seronegatives SS geäußert worden. Die Patientin berichtete über eine ausgeprägte Augentrockenheit seit 21 Jahren mit extremer Blendempfindlichkeit, der Ophthalmologe hatte eine Keratoconjunctivitis sicca festgestellt. Auch habe sie seit 2 Jahren eine trockene, photosensible Haut. In der klinischen Untersuchung zeigten sich die Augen stark blendempfindlich und die PD > 3 cm und die SMD bei 2 cm prominent palpabel.

### Beschreibung Patientin 4

Das primäre SS der heute 58-jährigen Patientin zeigte seit 2001 zunächst einen moderat aktiven, aber progredienten Verlauf mit dauerhaft geschwollenen PD und SMD, zervikaler Lymphadenopathie und ausgeprägter oraler, okulärer und vaginaler Sicca-Symptomatik. Die oralen Sicca-Symptome hatten 7 Jahre und die Parotisschwellungen 1 Jahr vor Erstdiagnose begonnen. Im Verlauf entwickelte die Patientin zudem eine leukozytoklastische Vaskulitis an beiden Unterschenkeln, Arthritis der proximalen Interphalangeal- und Kniegelenke, Myalgien, progrediente Fatigue mit B‑Symptomen, eine zunehmende rechtsseitige Parotisschwellung und einen Abfall des Ig(Immunglobulin)G von 31,1 g/l auf 12,4 g/l. Daher erfolgte neben einer SD-Sonographie auch eine Biopsie der rechten PD.

### Beschreibung Patientin 5

Erstmanifestation der Sarkoidose vor 32 Jahren akut mit Schwellungen beider Sprunggelenke und schmerzhaften rötlichen Knoten an den Unterschenkeln. Zudem habe eine bewegungslimitierende Schwellung des Halses bestanden. Besserung seinerzeit nach hoch dosierten Steroiden, die über 6 Jahre ausgeschlichen wurden. Die aktuelle Vorstellung erfolgte mit Arthralgien der großen und mittelgroßen Gelenke ohne Morgensteifigkeit und zunehmender Mund- und Augentrockenheit, Abgeschlagenheit, Gewichtsverlust und Nachtschweiß. Augentropfen wurden noch nicht angewendet. In der klinischen Untersuchung fanden sich die Tränen- und Speicheldrüsen prominent palpabel (PD bei < 3 cm). Zur weiteren Abklärung sonographierten wir die SD, quantifizierten Tränen- und Speichelfluss und stellten die Patientin ferner zur SDB in der HNO(Hals-Nasen-Ohren)-Klinik vor.

### Beschreibung Patient 6

Der 66-jährige Patient stellte sich ambulant vor mit okulärer und oraler Sicca-Symptomatik, Schwellungen der Tränendrüsen und einer massiv ausgeprägten Lymphadenopathie. Zudem bestanden Gewichtsabnahme, Fatigue, Reizhusten und Belastungsdyspnoe mit verminderter Belastbarkeit. In der klinischen Untersuchung fand sich neben der Lymphadenopathie und einer deutlichen SD-Vergrößerung (PD > 3 cm) auch eine Splenomegalie, die eine weitere Fokussuche nach sich zog.

Weitere Details zu den oben genannten Fällen finden sich in Tab. 1 und im Ergebnisteil.

**Table 1:** 

Fall	Alter	Geschlecht	ANA-Status	SS-A/SS-B-Ak-Status	Ultraschallergebnis	Schirmer-Test (LA/RA in mm)	Speichelflussrate unstimuliert/stimuliert (ml/min)	ESSDAI	Histologie	Diagnose nach Befunden	Therapieversuch
1	32	Weiblich	1:3400	+/+	PD/SMD: B‑Bild III°, CD I°	5/21	0,15/0,315	17	Speicheldrüsenparenchym mit zentral konfluierenden lymphoplasmazellulären und histiozytären Aggregaten	Primäres Sjögren-Syndrom	Hydroxychloroquin, MTX; hyaluronsäurehaltige Augentropfen; künstlicher Speichel
2	56	Weiblich	1:400	−/−	PD bds und rechte SMD deutlich vergrößert, B‑ und CD-Bild II° mit echoarmen Foci	14/35	0,63/0,88	2	Geringgradig fibrosiertes Speicheldrüsenparenchym ohne nennenswertes entzündliches Infiltrat	Sekundäres Sjögren-Syndrom bei undifferenzierter Kollagenose	Hydroxychloroquin; künstlicher Speichel
3	71	Weiblich	1:100	−/−	PD/SMD: blande	0/5	1,01/1,81	4	Minimale lymphoplasmazelluläre Infiltrate innerhalb fibrosierten Speicheldrüsenparenchyms	Keratoconjunctivitis sicca	Hyaluronsäurehaltige Augentropfen
4	58	Weiblich	1:12800	+/+	B‑Bild II°, CD 0°; 4 × 1 cm messende, aus mehreren echoreichen, runden Strukturen bestehende Raumforderung	0/0	0,01/0,04	28	In der Biopsie zunächst noduläre lymphoplasmazelluläre Aggregate mit fokaler Gangdestruktion Im Hemiparotidektomiepräparat rasenbildendes lymphozytäres Infiltrat mit lymphoepithelialen Läsionen	Primäres Sjögren-Syndrom; Marginalzonen-B-Zell-Lymphom auf dem Boden einer lymphoepithelialen Sialadenitis	Hydroxychloroquin; „off-label“ Rituximab; Pilocarpin; hyaluronsäurehaltige Augentropfen; Hemiparotidektomie
5	65	Weiblich	ANA negativ	−/−	Grießige, inhomogene Struktur des gesamten Areals der PD und SMD, aber ohne eindeutige echoarme Foci; B‑Bild 0°, CD 0°	7/4	0,72/0,95	Kommt nicht zur Anwendung	Ausgedehnte epitheloidzellige granulomatöse Entzündung mit Verdrängung des Speicheldrüsenparenchyms	Sarkoidose	Steroidstoß, MTX; zuckerfreie Kaugummis; Empfehlung hyaluronsäurehaltige Augentropfen
6	66	Männlich	ANA 1:200	Anti-PM-Scl+, RF 17 IE/ml, Eosinophile 10 %; IgG4 15.800 g/l	Vergrößerte, komplett mit echoarmen bis echofreien Strukturen durchbaute PD mit massiv gesteigerter Durchblutung	3/4	0,12/0,27	Kommt nicht zur Anwendung	Speicheldrüsenparenchym mit ausgedehnten IgG4-positiven plasmazellulär betonten Infiltraten mit Lymphfollikelbildung	IgG4-assoziierte Erkrankung	Steroidstoß, MTX; Empfehlung zuckerfreie Kaugummis; Empfehlung hyaluronsäurehaltige Augentropfen

*ANA* antinukleäre Antikörper, *Anti-PM-Scl , Ak* Antikörper *bds* beidseitig, *CD* Color-Doppler, *ESSDAI* EULAR (European League Against Rheumatism) Sjogrenʼs Syndrome Disease Activity Index, *Ig* Immunglobulin, *LA* linkes Auge, *MTX* Methotrexat, *PD* Parotisdrüsen, *RA* rechtes Auge, *RF* Rheumafaktor, *SMD* Submandibularisdrüsen

Alle Sonographien wurden mit einem Esaote@MyLab Twice (Esaote, Köln, Deutschland) mit einer 18-MHz-Linearschallkopfsonde durchgeführt. Für die Color-Doppler-Einstellungen wurde eine Frequenz von 8 MHz und eine Pulsrepetitionsfrequenz von 400 Hz für PD, bzw. 800 Hz für SMD gewählt, analog zu [10].

## Ergebnisse

Nach wie vor gibt es keine zugelassenen synthetischen oder biologischen DMARDs („disease-modifying anti-rheumatic drugs“) zur Behandlung des Sjögren-Syndroms. Bei der hier vorgestellten Fallserie soll es daher im Wesentlichen um die Diagnostik und weniger um die Diskussion von Therapien gehen. Zur Quantifizierung der Speichelflussraten wurden die Normwerte nach Siedek et al. zugrunde gelegt [15].

### Patientin 1

**Figure 1:**
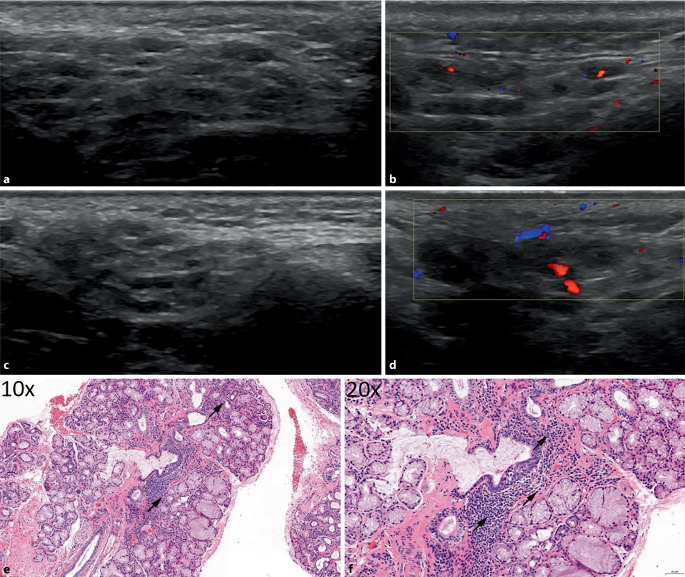

Der ANA(antinukleäre Antikörper)-Titer war stark erhöht, und es konnten Anti-SS-A-Antikörper (Ak) nachgewiesen werden. Sonographisch zeigten sich die PD sowie die SMD deutlich vergrößert mit hypoechogenen Foci im gesamten Drüsengewebe, entsprechend III°-Veränderungen sowie einer Hypervaskularisierung im Color-Doppler I° (entsprechend einer leichten Durchblutungssteigerung des Drüsengewebes; Abb. 1a–d). Im Rahmen eines Studienscreenings wurde außerdem eine SDB durchgeführt. Die Histologie ergab ein für ein primäres SS typisches Muster mit einer periduktal betonten lymphoplasmazellulären und histiozytären Infiltration mit teils konfluierenden Foci (Abb. 1e, f), sodass wir in der Zusammenschau aller Befunde den Verdacht auf primäres SS bestätigen konnten. Der Tränenfluss war leicht und die Speichelflussrate deutlich reduziert, sodass wir der Patientin eine symptomatische Therapie mit hyaluronsäurehaltigen Augentropfen und künstlichem Speichel empfahlen. Zudem erfolgte angesichts der hohen Krankheitsaktivität die Therapieeinleitung mit gewichtsadaptiertem Hydroxychloroquin und Methotrexat. Details s. Tab. 1.

### Patientin 2

**Figure 2:**
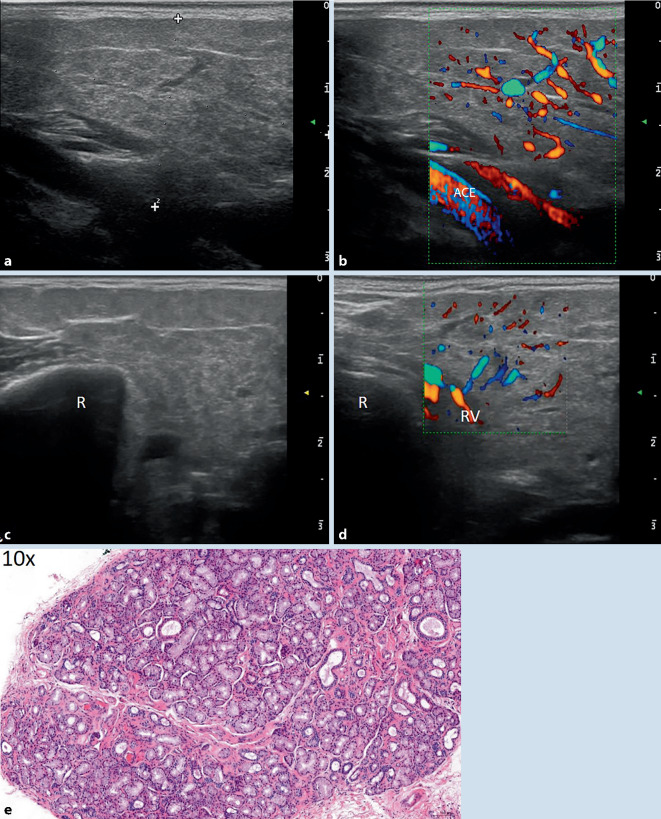

Der ANA-Titer war erhöht bei allerdings negativen Anti-SS-A-Antikörpern. Sonographisch waren beide PD und die rechte SMD deutlich vergrößert, im B‑ und CD-Bild fanden sich echoarme Foci entsprechend II°-Veränderungen bzw. einer Durchblutungssteigerung von < 50 % des Drüsengewebes (Abb. 2a–d). In der Histologie zeigte sich gering fibrosiertes Speicheldrüsengewebe ohne nennenswerte lymphoplasmazelluläre oder histiozytäre Infiltration (Abb. 2e). Der Tränenfluss und der unstimulierte Speichelfluss waren normwertig, der stimulierte Speichelfluss war leicht eingeschränkt. Wir diagnostizierten ein sekundäres Sjögren-Syndrom bei undifferenzierter Kollagenose. Die Patientin profitierte hinsichtlich ihrer intermittierenden Arthralgien von der Therapie mit Hydroxychloroquin und hinsichtlich ihrer Mundtrockenheit von der Anwendung von künstlichem Speichel. Details s. Tab. 1.

### Patientin 3

**Figure 3:**
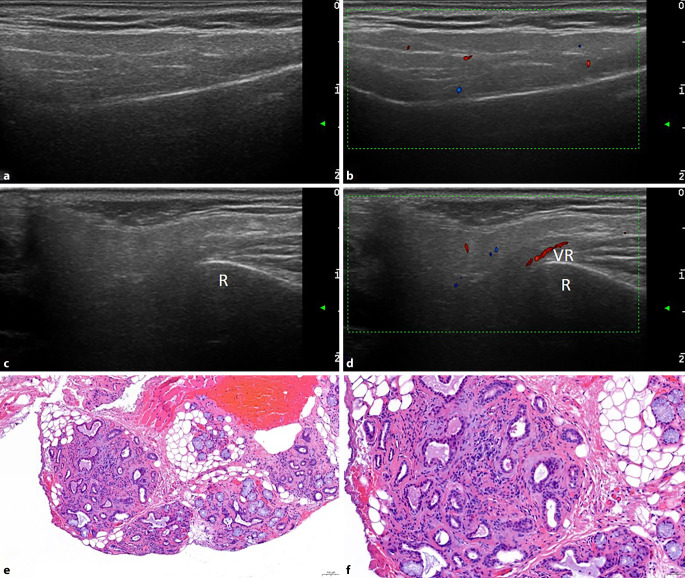

Der ANA-Titer war nur leicht erhöht, und die Anti-SS-A-Antikörper waren nicht nachweisbar. Der sonographische Befund ergab normal große und homogene Speicheldrüsen ohne Durchblutungssteigerung (Abb. 3a–d). In der Histologie zeigte sich multifokal fibrosiertes SD-Gewebe mit nur diskreter lymphoplasmazellulärer Infiltration (Abb. 3e, f). Der Speichelfluss war normal, der Tränenfluss jedoch reduziert, sodass wir in der Zusammenschau der Befunde die Keratoconjunctivitis sicca bestätigten, die topische Therapie mit hyaluronsäurehaltigen Augentropfen intensivierten und zu einer dauerhaften ophthalmologischen Anbindung rieten. Details s. Tab. 1.

### Patientin 4

**Figure 4:**
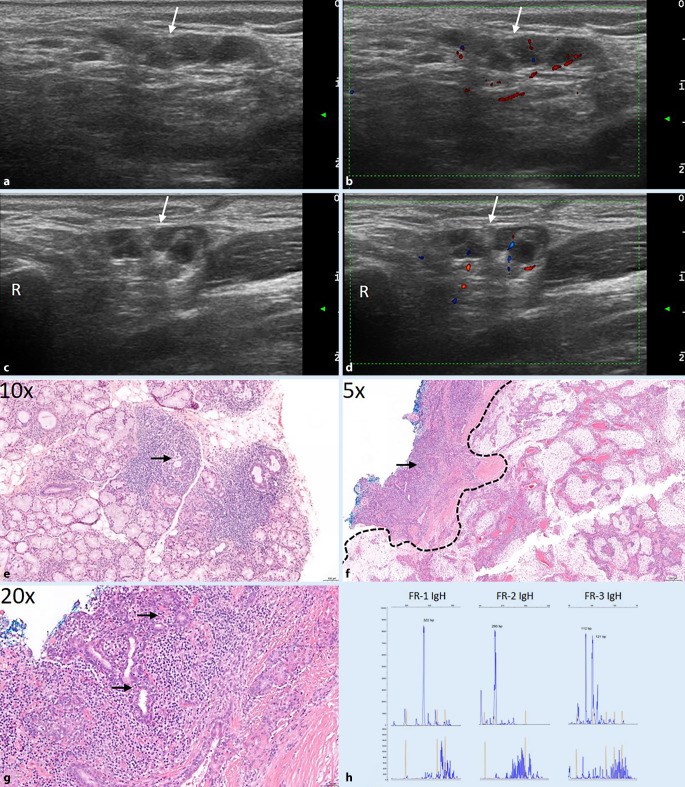

Bereits der hochpositive ANA-Titer, die positiven Anti-SS-A-Antikörper und die typische Klinik ließen seinerzeit das primäre SS vermuten. Dies wurde auch von dem initial typischen Ultraschallbefund (B-Bild II°) untermauert. Die initiale Histologie ergab vermehrte lymphoplasmazelluläre Infiltrate, die ebenfalls zu einem primären SS passten (Abb. 4e). Bei fokalem Nachweis von Gangdestruktion erfolgten ergänzende molekularpathologische Untersuchungen, in denen eine monoklonale B‑Zell-Population nachweisbar war. Da zu diesem Zeitpunkt jedoch die Kriterien eines Marginalzonenlymphoms nicht hinreichend erfüllt waren und in der Literatur auch monoklonale Befunde beim SS beschrieben sind, wurde lediglich eine Verlaufskontrolle empfohlen. Bei B‑Symptomatik und Nachweis einer Raumforderung von 4 × 1 cm, bestehend aus mehreren echoreichen, runden Strukturen (Abb. 4a–d), erfolgte eine Hemiparotidektomie, in der sich neben einem pleomorphen Adenom im umgebenden noch vorhandenen SD-Gewebe rasenbildende lymphozytäre Infiltrate teils mit lymphoepithelialen Läsionen zeigten (Abb. 4f, g). In Zusammenschau mit einem erneuten molekularpathologischen Nachweis einer B‑Zell-Klonalität (Abb. 4h) wurde ein initiales Marginalzonenlymphom diagnostiziert. Wir leiteten eine B‑Zell-depletierende Therapie mit „off-label“ Rituximab ein, das Hydroxychloroquin wurde fortgeführt, ebenso wie die Dauertherapie mit Pilocarpin und hyaluronsäurehaltigen Augentropfen. Gegenwärtig befindet sich die Patientin in klinischer Remission. Details s. Tab. 1.

### Patientin 5

**Figure 5:**
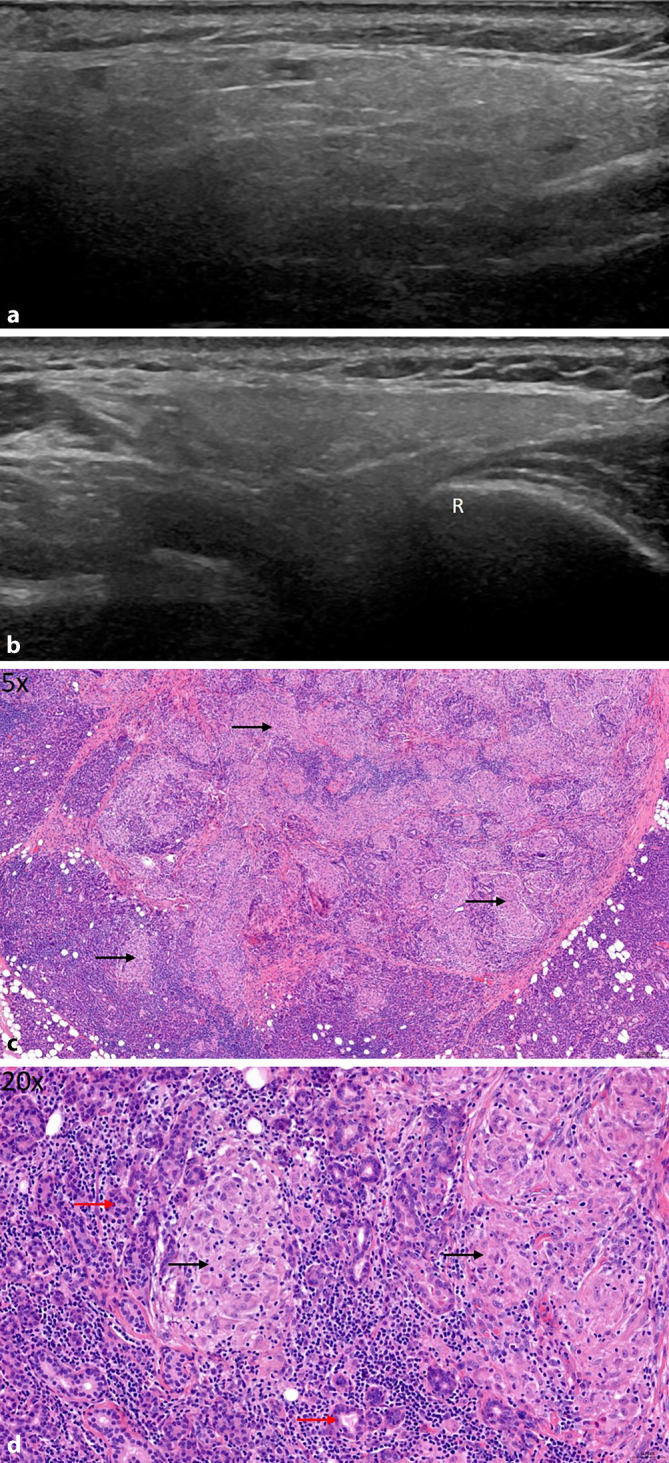

Auch bei dieser Patientin waren Klinik und Vorgeschichte diagnoseweisend; bestätigend waren ANA und Anti-SS-A-Antikörper nicht nachweisbar, Tränen- und Speichelfluss waren leicht vermindert. Sonographisch zeigte sich eine grießige, inhomogene Struktur des gesamten Areals der PD und SMD, aber ohne eindeutige echoarme Foci, entsprechend B‑ und CD-Mode 0° (Abb. 5a, b). Eine entsprechende Histologie zeigte eine ausgeprägte granulomatöse Entzündungsreaktion innerhalb der SMD. Nach Ausschluss weiterer Differenzialdiagnosen passte der Befund gut zur Verdachtsdiagnose einer Sarkoidose (Abb. 5c, d). Die Patientin sprach gut auf den Steroidstoß und die immunsuppressive Therapie mit Methotrexat an. Zur symptomatischen Behandlung der Mundtrockenheit genügten der Patientin zuckerfreie Kaugummis, zudem empfahlen wir die Anwendung hyaluronsäurehaltiger Augentropfen. Details s. Tab. 1.

### Patient 6

**Figure 6:**
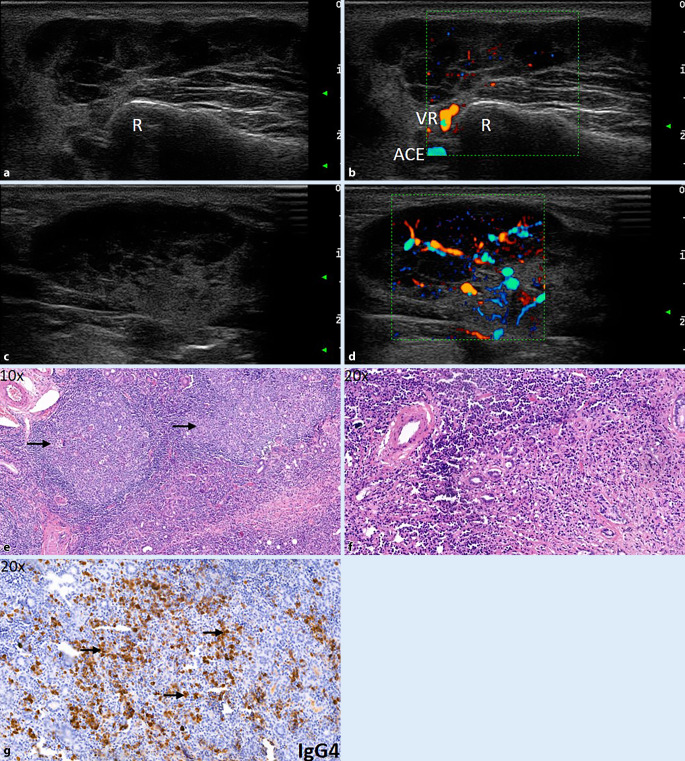

Der schlechte Allgemeinzustand des Patienten mit Belastungsdyspnoe und Splenomegalie veranlassten uns zu einer umfangreichen Diagnostik. Das immunologische Labor war nicht eindeutig, sodass IgG4 nachbestimmt wurde, das massiv erhöht war. Die SDB ergab vergrößerte, komplett mit echoarmen bis echofreien Strukturen durchbaute PD mit massiv gesteigerter Durchblutung, nicht typisch für ein SS (Abb. 6a–d). Die Lungenfunktion mit DLCO (Diffusionskapazität von Kohlenmonoxid) zeigte eine leichte Diffusionsstörung, die Thorax-CT (Computertomographie) milchglasartige Lungenparenchymveränderungen, sodass eine Immuno-BAL (bronchoalveoläre Lavage) erfolgte; hierin fanden sich 50 % Lymphozyten und ein CD4/CD8-Quotient von 8,6. Die SDB zeigte ein ausgeprägtes plasmazellulär betontes Infiltrat mit Follikelbildung. Bei der Frage nach einer IgG4-assoziierten Erkrankung erfolgten ergänzende immunhistochemische Untersuchungen, in denen sich der Großteil der Plasmazellen als IgG4 positiv darstellte (Abb. 6e–g). Wir leiteten einen Steroidstoß ein und begannen eine immunsuppressive Therapie mit Methotrexat, auf die der Patient allmählich ansprach. Details s. Tab. 1.

## Diskussion

In den hier vorgestellten Fallbeispielen zeigte sich, dass normalerweise Klinik und Serologie bereits erste Hinweise auf die zugrunde liegende Erkrankung geben. Bei nicht eindeutigen bzw. komplexen Fällen können jedoch Speicheldrüsenultraschall und Histologie wertvolle Informationen liefern, um die klinische Verdachtsdiagnose sichern und eine adäquate Therapie einleiten zu können. Beide Diagnostikverfahren sind dabei komplementär zu verstehen, um einen möglichst großen Nutzen für die Patient*innen zu erzielen.

Solange die SD-Sonographie jedoch noch nicht Einzug in die Diagnosekriterien gehalten hat, wird man noch nicht ganz auf Routinebiopsien verzichten können.

## Fazit für die Praxis

- Lange Zeit galt die Speicheldrüsen(SD)-Biopsie – besonders bei SS-A-negativen Patienten – als Diagnostik der Wahl, während der Stellenwert der SD-Sonographie auch heute noch kontrovers diskutiert wird.
- Der Ultraschall der Speicheldrüsen ist eine schnell durchführbare und nichtinvasive Methode, Sjögren-Syndrom(SS)-typische Veränderungen der großen SD zu detektieren und semiquantitativ einzuschätzen.
- Sowohl der Speicheldrüsenultraschall als auch die histologische Aufarbeitung der Speicheldrüsenbiopsien haben eine Berechtigung in der SS-Diagnostik und Differenzialdiagnose des Sicca-Syndroms. Beide Diagnostikverfahren sind komplementär zu verstehen, um einen möglichst großen Nutzen für die Patient*innen zu erzielen.
